# Dietary supplementation with *α*-linolenic acid improves the reproductive performance of blue foxes (*Alopex lagopus*)

**DOI:** 10.3389/fvets.2026.1772039

**Published:** 2026-05-13

**Authors:** Chongshan Yuan, Xingyuan Zhu, Wenhui Cao, Lei Sun, Shuai Qi, Jing Li, Jia Wang, Xinyan Cao

**Affiliations:** College of Animal Science and Technology, Jilin Agricultural University, Changchun, Jilin, China

**Keywords:** antioxidant, blue fox, semen quality, testicular tissue, *α*-linolenic acid

## Abstract

**Introduction:**

As a valuable fur-bearing species, enhancing the reproductive performance of male foxes is crucial for genetic advancement and productivity. *α*-Linolenic acid (ALA) has been reported to improve semen quality in male animals; however, its effects on reproductive parameters in blue foxes remain poorly characterized.

**Methods:**

In this study, 40 male blue foxes of similar age and condition were randomly assigned to four dietary groups (*n* = 10 per group) receiving a basal diet supplemented with 0, 0.5, 1.25, or 2 g/kg of ALA. Semen quality, antioxidant capacity, serum hormone levels, and testicular histomorphology were evaluated.

**Results:**

The results showed that compared with the control group (ALA, 0 g/kg), the 0.5 *α* group (ALA, 0.5g/kg), 1.25 α group (ALA, 1.25 g/kg), and 2 *α* group (ALA, 2 g/kg) significantly increased sperm motility, viability, sperm concentration, superoxide dismutase (SOD), catalase (CAT), testosterone (T), and follicle-stimulating hormone (FSH), as well as the perimeter and area of seminiferous tubules, while malondialdehyde (MDA) levels were significantly reduced (*p* < 0.05). Sperm abnormality rates were significantly lower in the 1.25 *α* and 2 *α* groups compared to 0 *α* group, along with elevated total antioxidant capacity (T-AOC), luteinizing hormone (LH), and sertoli cell counts (*p* < 0.05).

**Discussion:**

In conclusion, dietary ALA supplementation improves reproductive performance in male blue foxes by enhancing semen antioxidant capacity, modulating serum hormone profiles, and promoting testicular development, with an optimal supplementation level of 1.25 g/kg.

## 1 Introduction

As an economically important fur-bearing species, the blue fox (*Vulpes lagopus*) holds significant value in the fur farming industry, with its pelts prized for softness, desirable coloration, and superior thermal insulation (1). The blue fox breeding industry has achieved considerable scale in Northern Europe, Asia, and Africa, contributing substantially to regional economic development and employment (2). Genetically superior male blue foxes not only enhance conception rates but also improve the fur quality of their offspring, thereby increasing the overall economic returns of blue fox farming (3). Despite this economic importance, current understanding of reproductive physiology in blue foxes remains limited (4). Semen quality constitutes a fundamental determinant of reproductive performance, with high-quality semen being essential for ensuring satisfactory pregnancy rates, litter sizes, and offspring survival in female animals (5). Semen quality in male animals is influenced by multiple factors, including age, hormonal status, genetic background, and dietary nutrition. Among these, adequate nutritional intake through diet is crucial for maintaining optimal male fertility (6). However, while the regulation of semen quality through dietary nutrition has been extensively studied in livestock species, research on this topic in blue foxes remains conspicuously scarce, representing a notable gap in the current knowledge base.

The influence of nutrition on semen quality is depends on the calorie content in the diet, as well as specific characteristics of fatty acids, carbohydrates, and proteins (7). Among these, dietary fatty acids play a crucial role in maintaining male fertility due to their involvement in membrane fluidity, acrosome reaction, sperm motility, and viability (8). In many mammalian species, *ω*-3 polyunsaturated fatty acids (PUFAs) can constitute up to 60% of total sperm fatty acids (9). Since vertebrates cannot synthesize *ω*-3 PUFAs endogenously, these essential fatty acids must be obtained through dietary intake (10). As a member of the ω-3 PUFA family, *α*-linolenic acid (ALA) has been shown to contribute significantly to semen quality improvement (11). For instance, ALA supplementation has been reported to enhance semen quality, antioxidant capacity, and sperm fatty acid composition (12), while additionally reducing lipid peroxidation in semen and improving *in vivo* fertility outcomes in boars (13); similar benefits have been observed in bulls, where dietary ALA increases docosahexaenoic acid (DHA) content in sperm and improves frozen–thawed semen quality (14). Despite the well-documented role of ALA in supporting male fertility across several species, its effects on reproductive performance in blue foxes remain entirely unexplored.

This study evaluated the effects of dietary supplementation with different concentrations of ALA (0, 0.5, 1.25, and 2 g/kg) on reproductive parameters in male blue foxes. Parameters assessed included semen quality, antioxidant status, serum hormone levels, and testicular histomorphology. The objective was to identify the optimal ALA supplementation level for enhancing male fox fertility, thereby providing a theoretical basis for its application in breeding programs.

## 2 Materials and methods

### 2.1 Animals and experimental design

The study was conducted at Institute of Special Animal and Plant Sciences of Chinese Academy of Agricultural Sciences, Zuojia, Jilin Province. Forty healthy and similarly weighted fertile blue foxes (approximately 18 weeks of age) were selected and divided randomly into four groups. There were 10 blue foxes in the control group, and the remaining 30 were evenly divided into three experimental groups, with 10 foxes in each group. Each blue fox was kept in an individual cage (1.0 m × 1.0 m) and kept under similar management. According to the previous study (15), the 0 *α* group was fed basic feed, while the 0.5 α, 1.25 α, and 2 α group were supplemented with 0.5, 1.25, and 2 g/kg ALA (Jiangsu Baoshijia Biotechnology Co., Ltd. Jiangsu, China) in the basic diet, respectively. Composition and nutrient levels of basal diet are shown in Table 1. Feed was provided at 06:30 and 16:00, and water was provided *ad libitum*. After feeding ALA for 10 weeks, samples were collected.

**Table 1:** Composition and nutrient levels of basal diet (dry matter basis).

Ingredients	Content (%)	Nutrition	Content (%)
Extruded corn	25.05	Crude protein (CP)	28.02
Soybean meal	25.3	Ether extract (EE)	12.54
Bone and meat meal	8.25	Ash	7.29
Corn germ meal	20.75	Ca	1.28
Fish meal	9.2	P	1.02
Soybean oil	10	Methionine	0.49
NaCl	0.3	Lysine	1.66
Premix^a^	1		
Methionine	0.05		
Lysine	0.1		
Total	100		

^a^The premix contained per kg: Mixed vitamins 16 g; nicotinic acid 4000 mg; pantothenic acid 1200 mg; alkaloid 20 mg; folic acid 80 mg; choline 30,000 mg; Fe 8200 mg; Cu 800 mg; Mn 1200 mg; Zn 5200 mg; I 50 mg; Se 20 mg; Co 50 mg.

### 2.2 Sample collection

The semen ejaculates of the eighth week were collected by professional technicians using the gloved hand technique. Blood samples were collected from the hind leg vein into serum collection tubes and allowed to clot. Serum was separated after centrifugation at 2,500 rpm for 30 min. At the end of the trial, five blue foxes were randomly selected from each group and humanely slaughtered *via* electric shock according to the guidelines outlined in the Welfare of Animals Kept for Fur Production. Following euthanasia, the testicles of the blue foxes were collected and placed in 4% paraformaldehyde fixative (Sangon Biotech, Co., Ltd., Shanghai, China).

### 2.3 Semen quality analysis

Sperm motility was evaluated by placing 10 μL of undiluted semen on a pre-warmed (37 °C) slide and examining five random fields of view (≥ 200 sperm each) under a high-power optical microscope (400 ×), with straight-line motile sperm counted three times per sample. Sperm viability was assessed using the trypan blue staining method, where 10 μL of extended semen was mixed with an equal volume of staining solution (Sangon Biotech, Shanghai, China) and incubated at room temperature for 1–3 min; live sperm remained transparent and colorless, while dead sperm exhibited stained membranes. For sperm concentration, 10 μL of semen was diluted with 90 μL of physiological saline with 4% formalin, loaded into a hemocytometer, and sperm with all intact sperm heads were counted within 25 small squares (5 × 5) under high-power magnification. The sperm abnormality rate was determined using Giemsa staining, wherein 100 μL of extended sperm was fixed in 900 μL of 4% paraformaldehyde for 15 min, followed by staining with Giemsa solution (Sangon Biotech, Shanghai, China). For each parameter, five random fields of view were examined, each containing no less than 200 sperm, and all samples were analyzed in triplicate, with the average values reported.

### 2.4 Endogenous antioxidant indices in semen and hormone levels in serum

Seminal plasma was separated by centrifugation at 3,500 rpm for 15 min, while serum was obtained by centrifugation at 2,500 rpm for 30 min, with both samples subsequently stored in 1.5 mL centrifuge tubes. Endogenous antioxidant indices, including superoxide dismutase (SOD, U/mL), malondialdehyde (MDA, nmol/mL), total antioxidant capacity (T-AOC, mM), and catalase (CAT, U/mL), were determined using commercial colorimetric assay kits (Nanjing Jiancheng Technology Co., Ltd., Nanjing, China), following the manufacturer’s instructions. The serum hormone levels including testosterone (T, nmol/L), follicle-stimulating hormone (FSH, mIU/mL), and luteinizing hormone (LH, mIU/mL) were analyzed using commercial colorimetric assay kits (Shanghai Lengton Biotechnology Co., Ltd., Shanghai, China). For both assays, kits were equilibrated at room temperature for 30 min prior to detection. Samples, standards, or biotinylated antigen working solution (50 μL) were added to the respective wells, followed by incubation at 37 °C for 30 min. After five washes with PBST to remove unbound components, avidin-HRP (50 μL) was added and incubated at 37 °C for an additional 30 min. Following another five washes, the bound HRP catalyzed tetramethylbenzidine (TMB) to produce a colorimetric reaction, which was terminated by the addition of stop solution. Absorbance was measured at 450 nm using an enzyme-linked immunosorbent assay analyzer (Thermo Multiskan™ SkyHigh), with absorbance values inversely correlated with antigen concentrations in the samples.

### 2.5 Hematoxylin and eosin staining

After being fixed in 4% paraformaldehyde fixative for 24 h, 0.5–1 cm tissue segments were processed for paraffin embedding through a graded ethanol series: dehydration in 70% ethanol for 12 h, 80% ethanol for 12 h, 90% ethanol for 1 h, and two changes of absolute ethanol (1 h each). The tissues were placed in xylene for 10 min and embedded in paraffin wax. Serial sections of 5 μm thickness were prepared using a rotary microtome. Sections were mounted on glass slides, dried at 65 °C for 2 h, and deparaffinized through xylene (15 min) followed by rehydration in a descending ethanol series (absolute, 95, 80, 70, 50%—4 min each) and distilled water. Tissue sections were stained with hematoxylin for 10 min and counterstained with eosin for 20–30 s. After staining, sections were dehydrated through an ascending ethanol series (95%, absolute-2 min each) and cleared in xylene before being coverslipped with neutral resin. The morphology of testicular tissue was observed under 200 × and 400 × magnification. All histomorphometric analyses were performed using ImageJ (v1.53). For seminiferous tubule morphometry, at least 50 tubules with round cross-sections and intact basement membranes were analyzed per animal; tubules with artifacts or within 100 μm of the section edge were excluded. Tubular area and perimeter were measured after calibration. For cell counting, sertoli cells were identified by triangular nuclei with a prominent nucleolus adjacent to the basement membrane, with at least 30 tubules analyzed per animal. Leydig cells were identified by polygonal shape, eosinophilic cytoplasm, and round nuclei with nucleoli, counted in 10 random interstitial fields per animal at 400 × magnification.

### 2.6 Statistical analysis

All experiments employed a completely randomized design with the individual animal as the experimental unit. Animals were randomly assigned to groups (*n* = 10 per group for semen analysis; *n* = 5 per group for histology). Data are presented as mean ± SEM. *Post-hoc* power analysis (G*Power, *α* = 0.05) confirmed achieved power >0.90 for primary outcomes. One-way ANOVA with Tukey’s *post-hoc* test was performed using SPSS 27 (SPSS Inc., Chicago, IL, United States). Histomorphometric analyses were conducted blinded to group allocation. GraphPad Prism 9.5.0 (GraphPad Software, Inc., La Jolla, CA, United States) was used for figure preparation. Statistical significance was set at *p* < 0.05.

## 3 Results

### 3.1 Semen quality

**Figure 1:**
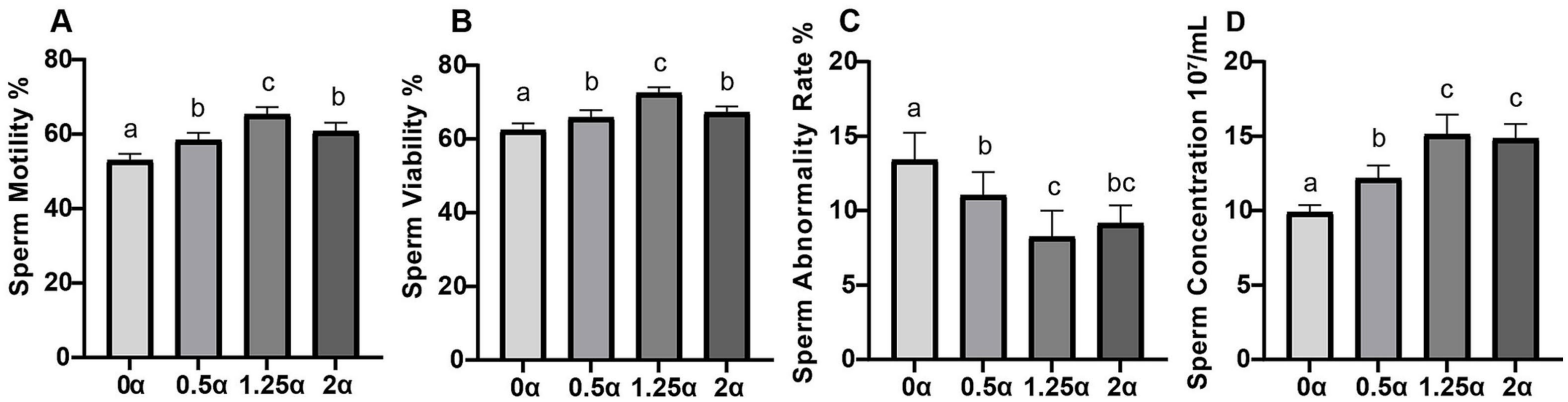
The effect of dietary supplementation with *α*-linolenic acid on semen quality of blue foxes. The supplementation levels of α-linolenic acid in the diet are 0, 0.5, 1.25, and 2 g/kg, respectively. **(A)** Sperm motility, **(B)** sperm viability, **(C)** sperm abnormality rate, **(D)** sperm concentration. Data are presented as means ± SEM. The same letter or no letter indicates no significant difference (*p* > 0.05), while different letters indicate significant differences (*p* < 0.05).

Figure 1 shows the effect of adding ALA to the diet on the quality of fresh semen in blue foxes. The present study revealed that adding 0.5, 1.25, and 2 g/kg ALA to the diet significantly improved sperm viability and motility compared with control group (*p* < 0.05). Compared with the 0 *α* group, supplementing with 1.25 g/kg and 2 g/kg ALA in the diet significantly reduced the sperm abnormality rate (*p* < 0.05). Among them, there was no significant difference in sperm abnormality rate between the 0.5 *α* group and the 0 α group (*p* > 0.05). Compared with the control group, the sperm concentration of the 0.5 α, 1.25 α, and 2 *α* groups was significantly increased (*p* < 0.05).

### 3.2 Endogenous antioxidant indices in semen

**Figure 2:**
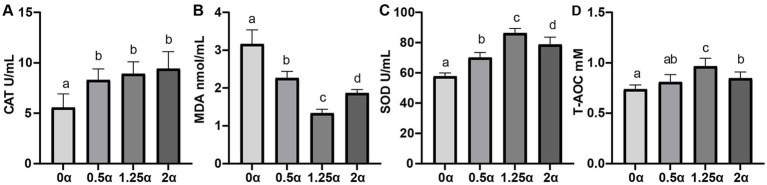
The effect of dietary supplementation with α-linolenic acid on semen endogenous antioxidant indices of blue foxes. The supplementation levels of α-linolenic acid in the diet are 0, 0.5, 1.25, and 2 g/kg, respectively. **(A)** Seminal plasma catalase (CAT, U/mL), **(B)** seminal plasma malondialdehyde (MDA, nmol/mL), **(C)** seminal plasma superoxide Dismutase (SOD, U/mL), **(D)** seminal plasma total antioxidant capacity (T-AOC, mM). Data are presented as means ± SEM. The same letter or no letter indicates no significant difference (*p* > 0.05), while different letters indicate significant differences (*p* < 0.05).

As shown in Figure 2, the concentration of T-AOC in the 1.25 *α* group was significantly increased compared with the control group (*p* < 0.05). Although there was a relative increased in T-AOC concentration between the 0.5 *α* group and the 2 α group compared to the 0 α group, there was no significant difference (*p* > 0.05). Compared with the 0 *α* group, the SOD activity levels of the 0.5 *α*, 1.25 α, and 2 α groups were significantly increased (*p* < 0.05), with the 1.25 α group having the highest level. Compared with the control group, adding different levels of ALA to the diet significantly reduced the concentration of MDA (*p* < 0.05). Compared with the 0 *α* group, the CAT activity content of the 0.5 *α*, 1.25 α, and 2 α groups were significantly increased (*p* < 0.05).

### 3.3 Hormones in serum

**Figure 3:**
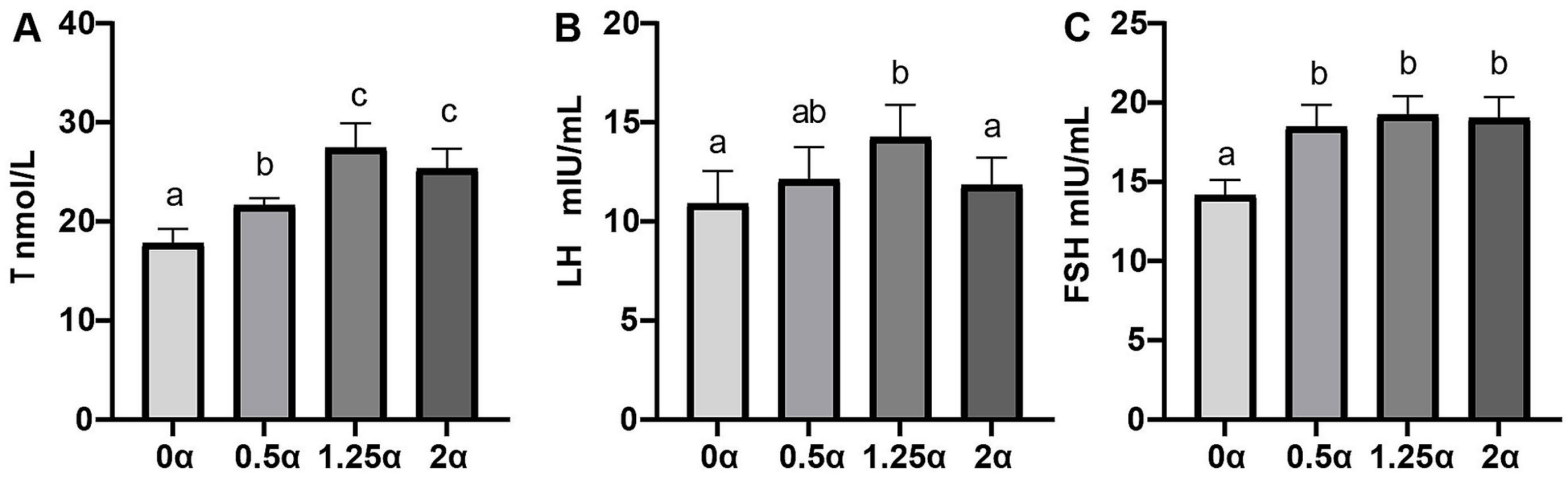
The effect of dietary supplementation with α-linolenic acid on serum hormones of blue foxes. The supplementation levels of α-linolenic acid in the diet are 0, 0.5, 1.25, and 2 g/kg, respectively. **(A)** Serum testosterone (T, nmol/L), **(B)** serum luteinizing hormone (LH, mIU/mL), **(C)** serum follicle stimulating hormone (FSH, mIU/mL). Data are presented as means ± SEM. The same letter or no letter indicates no significant difference (*p* > 0.05), while different letters indicate significant differences (*p* < 0.05).

The effect of dietary ALA on the hormone levels of serum is shown in Figure 3. Compare with the control group, supplementing different concentration of ALA significantly increased the levels of T (*p* < 0.05). Compared with the 0 *α* group, the LH level in the 1.25 α group was significantly increased (*p* < 0.05), while the other two experimental groups had no significant effect on the increase of LH level (*p* > 0.05). Meanwhile, compared with the 0 α group, the FSH levels in the 0.5 *α*, 1.25 α, and 2 α groups were significantly increased (*p* < 0.05). There was no significant difference in the effects among the three experimental groups (*p* > 0.05).

### 3.4 Testicular histomorphology

**Figure 4:**
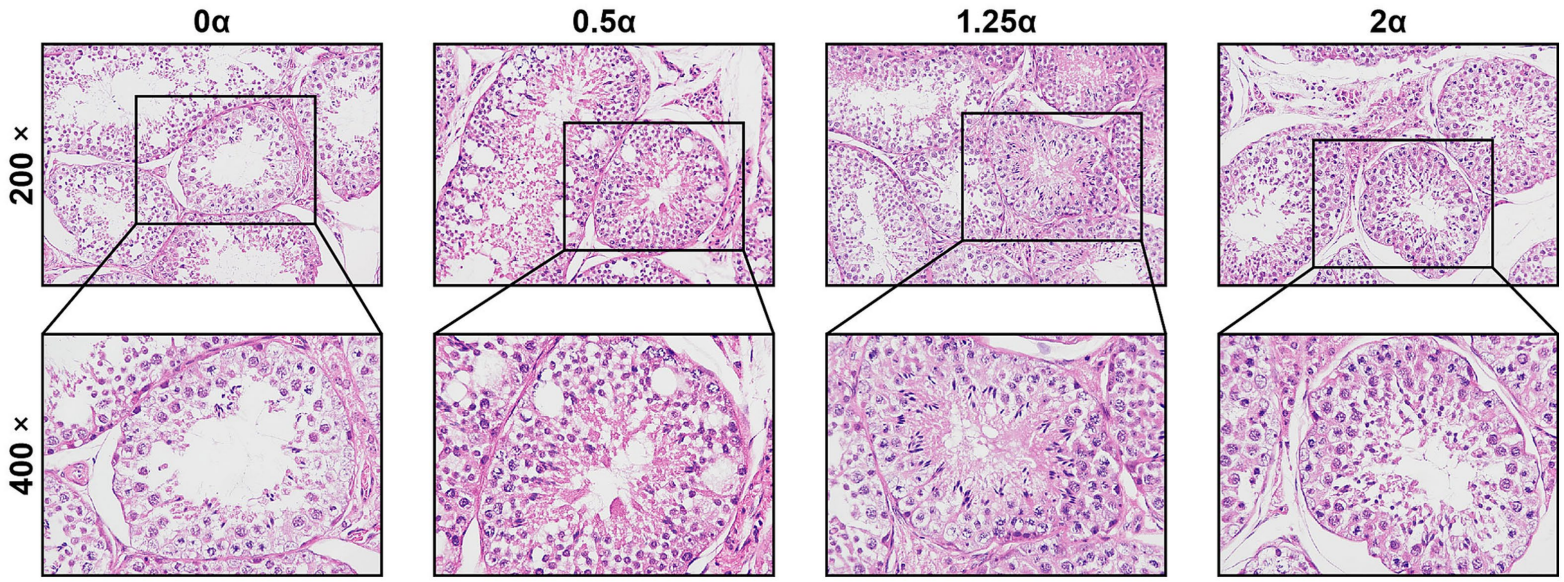
The effect of dietary supplementation with α-linolenic acid on testicular histomorphology of blue foxes. The supplementation levels of α-linolenic acid in the diet are 0, 0.5, 1.25, and 2 g/kg, respectively. The morphology of testicular tissue was observed under 200 × and 400 × magnification.

**Figure 5:**
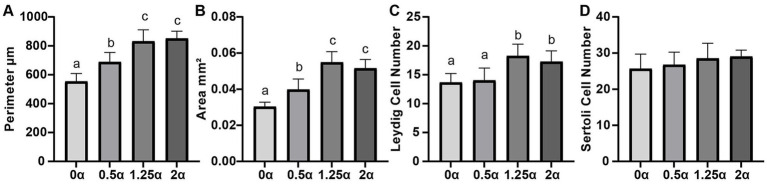
The effect of dietary supplementation with α-linolenic acid on the developmental pattern of testicles of blue foxes. The supplementation levels of α-linolenic acid in the diet are 0, 0.5, 1.25, and 2 g/kg, respectively. **(A)** Perimeter of seminiferous tubules, **(B)** area of seminiferous tubules, **(C)** number of leydig cells, **(D)** number of sertoli cells. Data are presented as means ± SEM. The same letter or no letter indicates no significant difference (*p* > 0.05), while different letters indicate significant differences (*p* < 0.05).

The effects of different dietary ALA levels on testicular tissue were evaluated using hematoxylin and eosin-stained histological sections (Figure 4). It can be seen from Figure 5 that compared with the 0 *α* group, dietary supplementation of 0.5, 1.25, and 2 g/kg ALA significantly increased the area of seminiferous tubules (*p* < 0.05). At the same time, the perimeter of the seminiferous tubules were also significantly increased among the experimental groups compared with the 0 α group (*p* < 0.05). Compared with the 0 α group, the number of leydig cells in the 1.25 α and 2 α groups were significantly increased (*p* < 0.05). There was no significant difference in the number of sertoli cells between the groups (*p* > 0.05).

## 4 Discussion

As an economically important fur-bearing species, the reproductive performance of male blue foxes significantly influences the economic sustainability of fur farming operations. While ALA, an *ω*-3 PUFA, has been shown to enhance semen quality in various livestock species, its effects on reproductive parameters in blue foxes remain unexplored. The present study evaluated the impact of dietary ALA supplementation at varying levels on semen quality, antioxidant status, serum hormone profiles, and testicular histomorphology in male blue foxes. Our findings demonstrate that ALA supplementation effectively improves semen quality, enhances testicular development, and strengthens antioxidant capacity in this species.

In the present study, dietary supplementation with ALA significantly enhanced sperm motility, viability, and concentration, while reducing the sperm abnormality rate in blue foxes. These findings aligns with previous reports in other species, including roosters (15) and bulls (14, 16–18), where ALA supplementation improved semen quality parameters. The beneficial effects of ALA on sperm quality are thought to be mediated in part through modifications in sperm membrane fatty acid composition, which influences membrane fluidity and functional integrity (14, 16). In the present study, the observed reduction in sperm abnormality rate may be associated with improved acrosome integrity, a relationship previously reported in other species (19). Additionally, the antioxidant properties of ALA may contribute to the preservation of sperm quality by mitigating oxidative stress-induced damage (20). While these mechanisms are supported by findings in livestock and poultry, direct evidence in blue foxes including confirmation of ALA incorporation into sperm membranes and its downstream metabolic conversion remains to be established.

Oxidative stress is recognized as a major contributor to male infertility, inducing sperm dysfunction and cellular damage through reactive oxygen species (ROS)-mediated pathways (21). Sperm membranes are rich in PUFAs, rendering them particularly vulnerable to ROS attack, which promotes lipid peroxidation and elevates MDA levels. T-AOC serves as a key indicator of redox homeostasis, with decreased T-AOC reflecting an imbalance between antioxidant defense and ROS generation that may lead to oxidative damage of cellular components (22). In the present study, ALA supplementation significantly reduced testicular MDA levels and increased total antioxidant capacity (T-AOC), consistent with its reported antioxidant properties in other species (23–26). Additionally, ALA supplementation enhanced the activities of superoxide dismutase (SOD) and catalase (CAT), two key enzymatic antioxidants that work cooperatively to maintain redox homeostasis (27, 28). Collectively, these findings suggest that ALA may improve semen quality in blue foxes, at least in part, by enhancing testicular antioxidant capacity and mitigating oxidative stress. However, direct evidence of ALA’s radical-scavenging activity and its effects on redox-regulating pathways in blue fox testicular tissue was not obtained in this study and warrants further investigation.

In addition to its antioxidant properties, reproductive hormones function as core regulators of the hypothalamic–pituitary-testicular (HPT) axis, playing a crucial role in maintaining the spermatogenic microenvironment and determining semen quality (29). Previous studies indicate that *ω*-3 PUFAs can modulate the secretion of hypothalamic gonadotropin-releasing hormone, pituitary gonadotropin release, and gonadal steroidogenesis (30). Our results corroborate these findings, demonstrating that ALA promotes the secretion of key reproductive hormones, including T, FSH, and LH. FSH supports spermatogenesis by acting on sertoli cells, while LH stimulates T production by leydig cells (29, 31). The observed increases in sperm concentration and quality following ALA supplementation coincided with elevated FSH and T levels, consistent with the established roles of these hormones in spermatogenesis (32). However, because we did not directly assess steroidogenic enzyme expression, gonadotropin receptor levels, or other molecular markers of HPT axis function, the precise mechanisms underlying these hormonal changes remain speculative. Future studies incorporating these molecular analyses are needed to establish whether ALA directly modulates the HPT axis or exerts its endocrine effects through indirect pathways.

The testes serve as the central organ for spermatogenesis, a process dependent on the specialized architecture of the seminiferous tubules (33). These tubules contain sertoli cells, which provide structural and nutritional support for germ cell development (34, 35), while leydig cells located in the interstitial compartment produce testosterone in response to luteinizing hormone (36, 37). In the present study, dietary ALA supplementation was associated with increased seminiferous tubule perimeter and cross-sectional area—key morphometric parameters reflecting spermatogenic capacity (38, 39). Additionally, leydig cell counts were significantly elevated, coinciding with increased serum testosterone levels. Although sertoli cell numbers did not reach statistical significance, an upward trend was observed. Collectively, these morphological changes are consistent with the improved sperm quality parameters reported above. However, the present study did not establish a direct causal relationship between ALA supplementation and these histological improvements.

## 5 Conclusion

In summary, dietary supplementation with ALA in male blue foxes enhances semen quality parameters-including motility, viability, and concentration-while reducing the sperm abnormality rate. These improvements are mediated through the modulation of testicular reproductive function, serum hormone profiles, and seminal plasma antioxidant capacity. The results demonstrate that ALA supplementation effectively promotes reproductive performance in male foxes, with an optimal dietary inclusion level of 1.25 g/kg.

## Data Availability

The original contributions presented in the study are included in the article/supplementary material, further inquiries can be directed to the corresponding authors.
